# The Amyloid-beta rich CNS environment alters myeloid cell functionality independent of their origin

**DOI:** 10.1038/s41598-020-63989-3

**Published:** 2020-04-28

**Authors:** Natalia Drost, Judith Houtman, Zoltán Cseresnyés, Raluca Niesner, Jan-Leo Rinnenthal, Kelly R. Miller, Stefan Prokop, Frank L. Heppner

**Affiliations:** 1 Department of Neuropathology, Charité – Universitätsmedizin Berlin, corporate member of Freie Universität Berlin, Humboldt-Universität zu Berlin, and Berlin Institute of Health, 10117 Berlin, Germany; 2 Present Address: German Center for Neurodegenerative Diseases (DZNE) Dresden, 01307 Dresden, Germany; 3 Deutsches Rheuma-Forschungszentrum Berlin, a Leibniz Institute, Charitéplatz 1, 10117 Berlin, Germany; 4 Present Address: Applied Systems Biology, Leibniz Institute for Natural Product Research and Infection Biology – Hans Knöll Institute, Jena, Germany; 5 Veterinary Medicine, Freie Universität, Berlin, Oertzenweg 19b, 14163 Berlin, Germany; 6 Present Address: Department of Pathology, Sana Klinikum Offenbach, 63069 Offenbach, Germany; 7 Present Address: Nanostring Technologies, Seattle, WA USA; 8 Present Address: Department of Pathology, University of Florida, Gainesville, FL United States; 9 Present Address: Center for Translational Research in Neurodegenerative Disease, University of Florida, Gainesville, FL United States; 10 Present Address: Fixel Institute for Neurological Diseases, University of Florida, Gainesville, FL United States; 11 Cluster of Excellence, NeuroCure, Charitéplatz 1, 10117 Berlin, Germany; 12 Berlin Institute of Health (BIH), 10117 Berlin, Germany; 13 German Center for Neurodegenerative Diseases (DZNE) Berlin, 10117 Berlin, Germany

**Keywords:** Alzheimer's disease, Microglia

## Abstract

Microglia, the innate immune cells of the central nervous system (CNS) survey their surroundings with their cytoplasmic processes, phagocytose debris and rapidly respond to injury. These functions are affected by the presence of beta-Amyloid (Aβ) deposits, hallmark lesions of Alzheimer’s disease (AD). We recently demonstrated that exchanging functionally altered endogenous microglia with peripheral myeloid cells did not change Aβ-burden in a mouse model mimicking aspects of AD at baseline, and only mildly reduced Aβ plaques upon stimulation. To better characterize these different myeloid cell populations, we used long-term *in vivo* 2-photon microscopy to compare morphology and basic functional parameters of brain populating peripherally-derived myeloid cells and endogenous microglia. While peripherally-derived myeloid cells exhibited increased process movement in the non-diseased brain, the Aβ rich environment in an AD-like mouse model, which induced an alteration of surveillance functions in endogenous microglia, also restricted functional characteristics and response to CNS injury of newly recruited peripherally-derived myeloid cells. Our data demonstrate that the Aβ rich brain environment alters the functional characteristics of endogenous microglia as well as newly recruited peripheral myeloid cells, which has implications for the role of myeloid cells in disease and the utilization of these cells in Alzheimer’s disease therapy.

## Introduction

Dementia caused by Alzheimer’s disease (AD) currently affects over 35 million people worldwide^1^ and is characterized by extracellular deposition of beta-Amyloid (Aβ), intracellular neurofibrillary Tau tangles and neurodegeneration^2,3^. The identification of microglia associated genes as risk factors for sporadic AD has highlighted the importance of microglia, the intrinsic immune cells of the central nervous system (CNS), in the disease process^4,5^. In the healthy brain microglia are surveying their environment with their fine, arborizing cytoplasmic processes, screening the entire brain parenchyma every few hours and rapidly responding to injury^6^. As a resident cell population with limited turnover^7^ microglia functionality as measured by baseline motility of cytoplasmic processes and response to injury declines with age^8^. We have recently shown that this phenomenon is accelerated and enhanced by the presence of Aβ-deposits in mouse models of AD-like pathology^9^. Increasing deposition of Aβ results in microglia priming, reduces microglia process movement, phagocytic capacity and impairs response to injury in two mouse models of AD-like pathology^9^. Manipulating this primed state of microglia by reducing pro-inflammatory cytokine signalling^10,11^ can reduce Aβ-deposits and improve cognitive decline, while increased microglia activation, even when going along with reduced Aβ-pathology^12–15^, can exacerbate associated pathology^16,17^.

Peripherally-derived myeloid cells (PDMCs) can enter the central nervous system (CNS) and contribute to the immune response in various brain disorders^18,19^. Although their contribution to the innate immune response in AD is limited^20–22^ it was suggested that enhanced recruitment of activated PDMCs can restrict pathology in AD-like mouse models^23^. We recently demonstrated that depletion of endogenous microglia in CD11b-HSVTK (TK)-mice^24^ is followed by rapid repopulation by PDMCs that adopt a microglia-like morphology^25^. This exchange of endogenous microglia, however, did not alter Aβ-pathology in two different AD-like mouse models^25,26^. Newly recruited PDMCs were initially not found to associate with Aβ plaques and only slowly surrounded plaques after residing in the CNS for over 3 months^26^. Despite not affecting the Aβ-burden at baseline, stimulation of these cells either by IgG or by Aβ-specific antibodies was able to mildly reduce Aβ-pathology^25^, indicating that PDMCs are impaired in a similar fashion to endogenous microglia in an Aβ-rich environment, but appear to be more responsive to activating triggers.

To better understand functional differences and similarities between these newly recruited PDMCs and endogenous microglia, we analysed morphology and functional parameters of microglia and PDMCs in wild type mice and a mouse model with AD-like Aβ-pathology using long-term 2-photon intravital microscopy, tracking the respective cell population over several weeks. By analysing myeloid cells in the myeloid cell exchange model recently established by us^24,25^ we were able to demonstrate that PDMCs exhibit smaller soma, shorter processes and showed a slightly faster response to a focal laser lesion than endogenous microglia. However, in an environment with amyloid pathology in AD-like mice, PDMCs acquired an altered functionality similar to resident microglia, especially near Aβ plaques.

## Results

### Peripherally-derived myeloid cells rapidly repopulate the microglia-depleted brain and adopt a microglia-like phenotype in wild type mice

**Figure 1:**
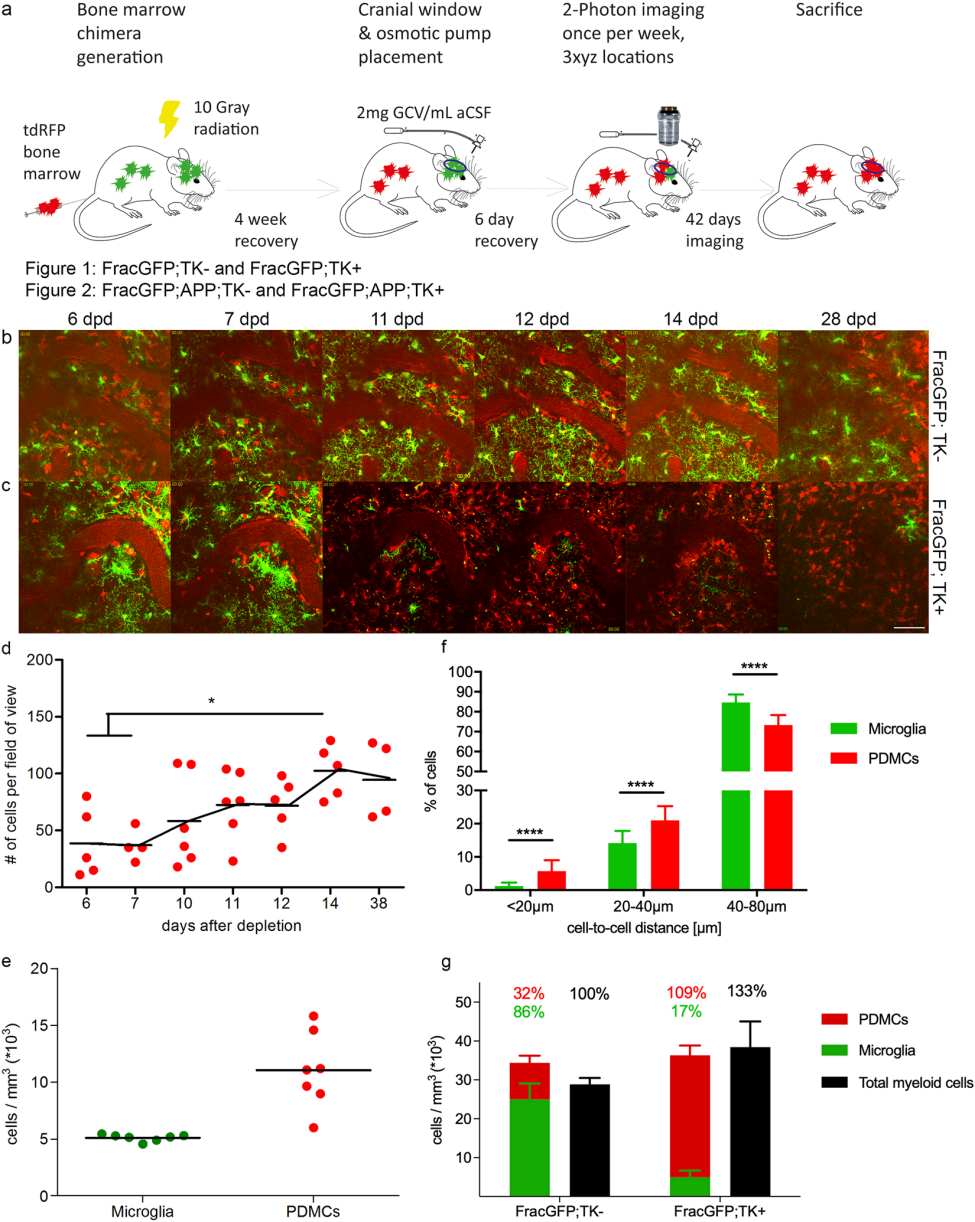
Peripherally-derived myeloid cells rapidly repopulate the microglia-depleted brain and adopt a microglia-like phenotype in non-AD mice. (**a**) Experimental time line. Mice were irradiated, and injected with tdRFP bone marrow cells. Four weeks after BM-transfer, a cranial window was installed and a mini-osmotic pump was implanted to deliver Ganciclovir for microglia ablation in TK+ animals. Imaging using 2-photon microscopy was started six days after surgery, and the mice were subsequently imaged once a week for six weeks. Mouse graphic designed by Gwilz [CC BY-SA 4.0 (https://creativecommons.org/licenses/by-sa/4.0)], from Wikimedia Commons. (**b,c**) Representative pictures from 2-photon imaging sessions displaying Frac-GFP;TK− (**b**) and Frac-GFP;TK+ (**c**) mice at indicated time points after surgery. GFP-positive cells represent endogenous microglia and RFP-positive cells represent peripherally-derived myeloid cells (PDMCs); scale bar = 100 µm. (**d**) Number of PDMCs per field of view over time; n = 6; degrees of freedom (df) = 34; 1-way ANOVA with Tukey post-hoc test, *p < 0.05. (**e**) Post mortem stereological quantification of microglia (FracGFP;TK−) and PDMC (FracGFP;TK+) cell density per mm^3^; n = 7; df = 12; Unpaired t-test ns. (**f**) Cell-to-cell distance of endogenous microglia (green bars) and PDMCs (red bars). Each field of view of the first minute of each imaging session was analysed in Imaris with the spot recognition algorithm. The xyz coordinates of spots were exported and the Euclidian distances between cells were measured for every detected cell with a custom written algorithm; n = 6, 3 fields of view per animal; df = 426; 2-way ANOVA with Sidaks post-hoc test; interaction <0.0001; ****p < 0.0001 (**g**) Distribution of PDMCs and microglia relative to total Iba1+ cells based on post mortem stereological quantification; FracGFP;TK− n = 3, FracGFP;TK+ n = 4.

To examine morphology and functionality of endogenous microglia and PDMCs in the non-diseased brain environment we crossed CX_3_CR_1_-GFP^+/−^ (Frac-GFP) males to CD11b-HSVTK^+/−^ (TK+) females. The resulting Frac-GFP;TK+ experimental mice were lethally irradiated and reconstituted intravenously with 10^7^ bone marrow (BM) cells isolated from ROSA26-tandem red fluorescent protein (tdRFP) reporter mice^27^, to allow distinction of endogenous microglia (GFP-positive) and PDMCs (RFP-positive) with *in vivo* 2-photon imaging in the context of microglia depletion and myeloid cell repopulation. Four weeks after the BM transfer, microglia depletion was initiated by implanting a mini-osmotic pump (model 2001 cat.no. 0000292, Alzet) containing 2.5 mg Ganciclovir (Cymevene®)/ml (hereafter referred to as GCV) into the right lateral ventricle as previously described^28^. During the same surgery, a cranial window was placed over the left hemisphere, as previously described^29,30^, surrounded by a custom-made titanium ring for 2-photon imaging. After 10 days (end of microglia depletion phase), the mini-osmotic pump (model 2001) was replaced by a long-term mini-osmotic pump (model 2004 cat.no. 0000298, Alzet), without disturbing the cannula to allow for maintenance of continuous solvent flow. Imaging was started six days after implantation of the 2001 pump model and the mice were imaged once a week for 6 weeks (experimental time line see Fig. 1a).

Frac-GFP;TK− control mice showed a moderate influx of RFP tagged PDMCs (Fig. 1b,g), not influencing homeostatic microglia morphology (Fig. S1a,b and Supplementary Movie 1 and 2). In Frac-GFP;TK+ mice we observed a progressive depletion of GFP-positive endogenous microglia, and already at day 6 of GCV treatment the first infiltrating RFP-positive peripheral myeloid cells are visible, which constituted the majority of the myeloid cell population at day 12 and their numbers kept increasing until day 14 post depletion, appearing to reach a plateau at this time point (Fig. 1c,d). The increased number of PDMCs populating the brain in Frac-GFP;TK+ mice (Fig. 1e) can be accounted for by the reduced cell-to-cell distance of these cells when compared to endogenous microglia (Fig. 1f).

Following the last imaging session, the mice were sacrificed, the brain was removed and histologically analysed. Stereological quantification confirmed the impressions described above. While in Frac-GFP;TK− control mice about 1/3 of Iba1 positive cells were peripherally derived (32%, Fig. 1g), Frac-GFP;TK+ showed a significant depletion of endogenous (GFP-positive) microglia, which were replaced by PDMCs (RFP-positive, Fig. 1g). The relatively high number of infiltrating PDMCs in the non microglia-depleted brain of Frac-GFP;TK− may be accounted for by some leakiness in the blood brain barrier (BBB) due to irradiation^18,31^. Varvel *et al*. also showed leakiness after pump implantation surgery and subsequent depletion of microglia^32^ although microglia depletion by systemic GCV treatment without pump implantation surgery did not cause a leaky BBB^24^. These infiltrating cells were shown to vanish and not contribute to the resident myeloid cell pool when microglia are present^33^. The overall number of Iba1-positive cells increased in the microglia-depleted and repopulated mice, as previously described^25^ (Fig. 1e,g). The PDMCs distributed equally over the brain, in a similar fashion as the endogenous microglia (Fig. S1c).

In summary, these findings demonstrate that PDMCs repopulate the microglia-depleted CNS rapidly and evenly distribute throughout the CNS parenchyma.

### *In vivo* imaging of the replacement of endogenous microglia by peripherally-derived myeloid cells in an AD-like environment

To study the different CNS populating myeloid cell populations in an AD-like environment, we crossed APPPS1^+/−^ CD11b-HSVTK^+/−^ mice to CX3CR1-GFP^+/−^ mice to generate CX3CR1-GFP^+/−^APPPS1^+/−^CD11b-HSVTK^+/−^ mice (Frac-GFP;APP+;TK+). Frac-GFP;APP+;TK− littermates served as control mice. APPPS1 mice show first Aβ deposits in the cortex already after 60 days. The pathology develops rapidly, spreading through the cortex and hippocampus, reaching a plateau after eight months (250 days). At this age they develop behavioural deficits as well^34^. The imaging period used in these experiments is set in the plateau phases of the disease, from ~160 to ~210 days of age.

As described above, the experimental mice were lethally irradiated and reconstituted with 10^7^ bone marrow cells isolated from tdRFP reporter mice^27^ and treated with GCV following the same time course as described above for Frac-GFP;TK+ mice (experimental time line see Fig. 1a). In addition, the AD-like mice were injected intraperitoneally (i.p.) with 10 mg/kg Methoxy X0_4_ to visualize Aβ plaques one day prior to each imaging session.

**Figure 2:**
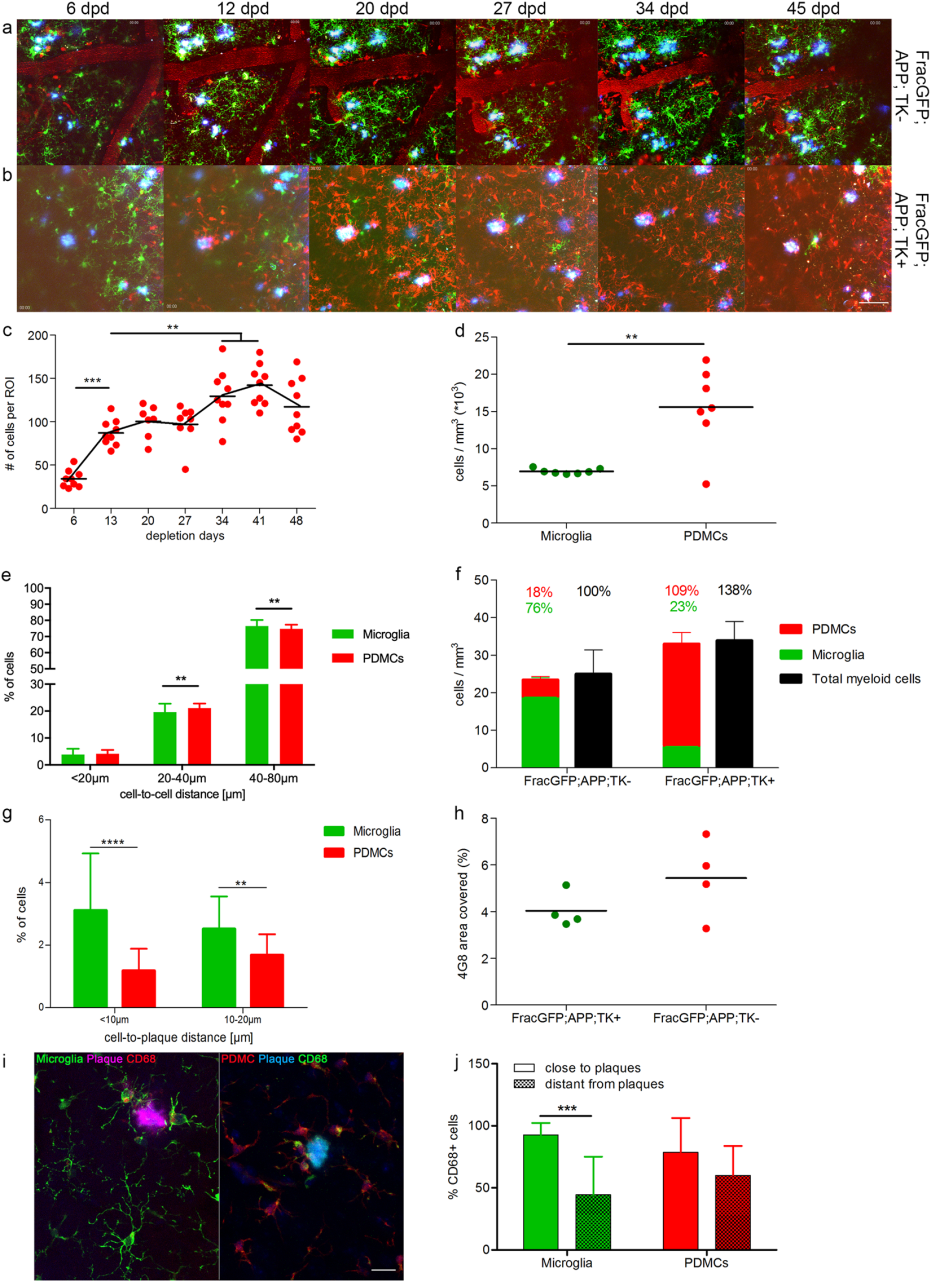
*In vivo* imaging of the replacement of endogenous microglia by peripherally-derived myeloid cells in an AD-like environment. (**a,b**) Representative images of 2-photon imaging sessions, for Frac-GFP;APP+;TK− (**a**) and Frac-GFP;APP+;TK+ (**b**) mice at indicated time points after surgery. GFP-positive cells represent endogenous microglia and RFP-positive cells represent PDMCs. Aβ plaques were stained by injecting Methoxy-X04 one day before each imaging session); scale bar = 100 µm. (**c**) Number of PDMCs per field of view over time; n = 7; df = 59; 1-way ANOVA with Tukey post-hoc test; **p < 0.01, ***p < 0.001. (**d**) Post mortem stereological quantification of microglia (FracGFP;TK−) and PDMC (FracGFP;TK+) cell density per mm^3^; n = 7; df = 12; Unpaired t-test; **p < 0.01. (**e**) Cell-to-cell distance of endogenous microglia (green bars) and PDMCs (red bars). Each field of view of the first minute of each imaging session was analysed in Imaris with the spot recognition algorithm. The xyz coordinates of spots were exported and the Euclidian distances between cells were measured for every detected cell with a custom written algorithm; n = 6, 3 fields of view per animal; df = 678; 2-way ANOVA with Sidak post-hoc test; interaction < 0.0001; **p < 0.01. (**f**) Distribution of PDMCs and microglia relative to total Iba1+ cells based on post mortem stereological quantification; n = 4. (**g**) Cell-to-plaque distance of endogenous microglia (green bars) and PDMCs (red bars). Each field of view of the first minute of each imaging session was analysed in Imaris with the spot recognition algorithm. The xyz coordinates of spots and plaques were exported and the Euclidian distances between cells and plaques were measured for every detected cell with a custom written algorithm; n = 6, 3 fields of view per animal; df = 327; 2-way ANOVA with Sidaks post-hoc test; interaction ns; **p < 0.01 ****p < 0.0001. (**h**) Morphometric analysis of area covered by 4G8 positive amyloid plaques; n = 4; Unpaired t-test ns. (**i**) maximum intensity projections of confocal stacks, showing a representative image of activated myeloid cells around plaques (distance < 10 µm) and less activated myeloid cells distant from the plaque; scale bar = 25 µm. (**j**) Quantification of the percentage of CD68 + activated myeloid cells close and distant from plaques; n = 3 animals, 4-6 fields of view per animal; 2-way ANOVA with Bonferroni post-hoc test; df = 57; interaction; ***p < 0.001.

Frac-GFP;APP+;TK− control mice showed a modest influx of PDMCs (Fig. 2a,f), similar to what we observed in non-AD Frac-GFP;TK− mice described above (see Fig. 1g). Microglia depletion in Frac-GFP;APP+;TK+ mice was followed by a rapid repopulation of the microglia-depleted CNS by PDMCs. Depletion of endogenous microglia was similarly effective for cells in vicinity of plaques (distance <10 µm) as for more homeostatic cells in further distance of plaques (Fig. 2b). The influx of PDMCs reached a first plateau around day 13 of imaging, similar to our observation in non-AD mice (Fig. 1c), and we noticed a continuous influx of peripheral cells in subsequent imaging sessions, albeit at slightly slower rate in the AD-like mice (Fig. 2c). The infiltrating cells were distributed evenly throughout the CNS parenchyma (Fig. S1d) and showed a difference in cell-to-cell distance (Fig. 2e), similar to the non-AD mice described above (Fig. 1f). We observed more endogenous microglia in close proximity to plaques when compared to PDMCs (Fig. 2g), in accordance with previously published data^25,26^. Independent of their origin, myeloid cells around plaques express high levels of CD68 as a marker of activation of phagocytic pathways (Fig. 2i,j). While endogenous microglia distant from plaques showed significantly less CD68 expression than endogenous microglia in close proximity of plaques (distance <10 µm), correlating with the morphological findings of a more homeostatic state of these cells described below, PDMCs in different distances from plaques did not exhibit this significant change in CD68 expression, indicative of a more macrophage-like phenotype of these cells (Fig. 2j).

Following the last imaging session, the brains of the experimental mice were analysed by stereology. Similar to the non-AD Frac-GFP;TK− mice described above (Fig. 1g), about 1/5 of Iba1 positive cells were peripherally derived (18%, Fig. 2f) in Frac-GFP;APP+;TK− control mice. In Frac-GFP;APP+;TK+mice, however, we observed a significant depletion of endogenous (GFP-positive) microglia, which were replaced by PDMCs (RFP-positive) consisting of about 4/5 of Iba1 positive cells (Fig. 2f). The overall number of Iba1-positive cells increased in the microglia-depleted and repopulated AD-mice (Fig. 2d), similar to what we observed in the non-AD setting described above (Fig. 1e). Furthermore, the exchange of endogenous microglia with PDMCs did not affect the overall Aβ-plaque burden in these mice (Fig. 2h), confirming previous reports using this model system^25,26^.

Since microglia depletion and repopulation follow a similar time course in AD-like and non-AD mice, we sought next to characterize myeloid cell morphology and function in detail, with specific emphasis on the relation of the myeloid cells to Aβ-deposits in the AD-like mouse model.

### Myeloid cells in the vicinity of Aβ plaques show altered morphology and function irrespective of their origin

**Figure 3:**
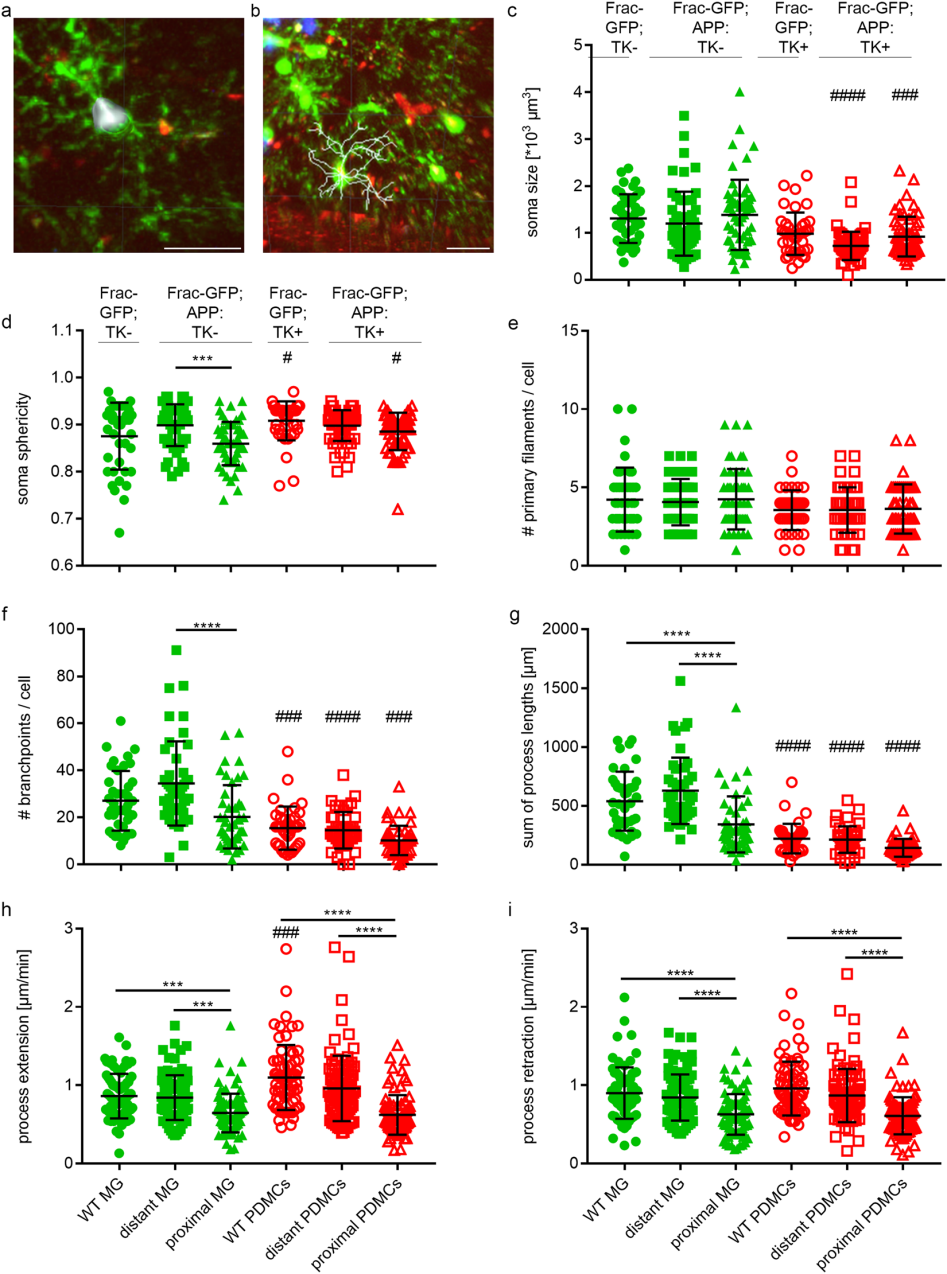
Myeloid cells in the vicinity of Aβ plaques irrespective of their origin show altered morphology and function. Morphological and functional parameters of endogenous microglia (green symbols) and PDMCs (red symbols) in both non-AD (Frac-GFP-TK− or Frac-GFP-TK+) and AD-like (Frac-GFP;APP+;TK− or Frac-GFP;APP+;TK+) mouse brain. Distant microglia and PDMCs are >10 µm distance from plaques, while proximal microglia and PDMCs are <10 µm distance from plaques. All recorded imaging videos were registered using the “Correct 3D drift” Plugin of ImageJ to correct for translational drifts^49^. Further analysis was performed in Imaris 7.0; Bitplane. (**a**) Example image of surface analysis by Imaris 7.0. The surfaces of cells were detected with the surface reconstruction algorithm of the software. All cell soma of one field of view were detected automatically in a batch, subsequently each movie was manually filtered for high quality detected somas); scale bar = 25 µm. (**b**) Example image of surface analysis by Imaris 7.0. The filament tree of the cells was reconstructed using the filament detection algorithm of Imaris for each cell individually; scale bar = 25 µm. (**c**) soma sphericity, mean values from the first minute of each imaging time point were used. 2-way ANOVA with Tukey’s post-hoc test; df = 307; no interaction. (**d**) soma size, mean values from the first minute of each imaging time point were used. 2-way ANOVA with Tukey’s post-hoc test; df = 307; interaction. (**e**) number of primary filaments. 2-way ANOVA with Tukey’s post-hoc test; df = 265; no interaction. (**f**) number of branchpoints 2-way ANOVA with Tukey’s post-hoc test; df = 255; interaction. (**g**) sum of process length. 2-way ANOVA with Tukey’s post-hoc test; df = 257; interaction. (**h**,**i**) Maximum intensity projections from the first minute and the fifth minute of each timepoint were analyzed. Five extensions and retractions of 2 up to 5 cells were randomly quantified as described before^6^ with ImageJ. (**h**) process extension speed. 2-way ANOVA with Tukey’s post-hoc test; df = 498; interaction. (**i**) process retraction speed. 2-way ANOVA with Tukey’s post-hoc test; df = 498; no interaction; (c-i) n = 3 animals, at 6 different time points, 1 dot represents the average values of 3 fields of view; ^*/#^p < 0.05, ^**/##^p < 0.01, ^***/###^p < 0.001, ^****/####^p < 0.0001. ^#^ denotes significant differences between the PDMCs and the corresponding microglia.

To get a quantitate assessment of the differences between endogenous microglia and PDMCs we used a basic set of parameters describing myeloid cell morphology and function (Fig. 3a). To capture the differences in myeloid cells dependent on their relation to Aβ plaques, we separated the myeloid cell populations in AD-like mice into myeloid cells in plaque proximity defined as cells in close proximity to the Aβ-deposits with their cell body or a process and myeloid cells distant from plaques, which did not have contact with the Aβ-deposits.

First, we analysed parameters describing the soma of the cells. The soma of PDMCs were significantly smaller than the soma of endogenous microglia in AD mice, independent of their relationship to Aβ plaques (Fig. 3b), indicating retention of some macrophage-like properties. The soma size increased over time in the non-AD setting, reflecting adaptation to the CNS environment and adoption of more microglia-like properties (Supplementary Fig. S2), which explains why the soma of the PDMCs in non-AD mice are not significantly different from the endogenous microglia at later stages. Soma size of PDMCs did not change over time in AD-like mice (Supplementary Fig. S2), indicating continuous stimulation and retention of macrophage-like properties in the presence of Aβ-deposits. The sphericity of the soma was similar between endogenous microglia and PDMCs in the plaque distance setting of AD-like mice. Endogenous microglia in proximity of Aβ plaques, however, showed increased soma sphericity compared to microglia distant from Aβ plaques (Fig. 3c) illustrating their activation and polarization towards Aβ-deposits with a more macrophage-like morphology. The fact that we did not see a similar change in soma sphericity in PDMCs in proximity to plaques when compared to PDMCs more distant from plaques indicates a somewhat reduced activation and polarization of PDMCs towards Aβ deposits.

Since microglia perform the surveillance of the CNS tissue mainly through systematic movement of their delicate and arborized processes, we next analysed the properties of cellular processes more closely.

While there was no difference in the number of primary filaments per cell between endogenous microglia and PDMCs, both in the non-AD and the AD setting and irrespective of the relation to plaques (Fig. 3d and Supplementary Fig. S3), we observed significant differences in the number of process branchpoints per cell and the sum of process length. These two parameters reflect the complexity and arborisation of the process tree. Endogenous microglia had more complex processes than PDMCs in non-AD mice as well as in AD-like mice. Interestingly, endogenous microglia in proximity of plaques had less branchpoints and a reduced sum of process length, making them more similar to PDMCs and reflecting a macrophage-like phenotype. These parameters did not significantly change during the observation period (Supplementary Fig. S3).

To get a more functionally relevant read-out of the cell processes, we assessed the extension and retraction speed of PDMCs. The shorter and less arborized processes of PDMCs showed faster extension and similar retraction speeds compared to the more complex processes of endogenous microglia in non-AD mice (Fig. 3g,h). However, both parameters displayed a significant reduction in process movement when endogenous microglia or PDMCs were in proximity of plaques (Fig. 3g,h). Plotting these parameters in single time points over the observation period revealed a significant decrease of process extension and retraction over time in PDMCs in the non-AD setting (Supplementary Fig. S4), again in line with adoption of a more microglia-like phenotype. There was some variation without an obvious trend over time in AD-like mice, irrespective of the distance to Aβ deposits, supporting the notion of retention of macrophage-like properties over time (Supplementary Fig. S4).

Quantitative analysis of multiple myeloid cell parameters demonstrated a non-homeostatic phenotype of plaque-associated microglia with shorter and less arborized processes, reduced sphericity of the cell soma and slower process movement when compared to endogenous microglia distant to plaques. PDMCs repopulating the microglia-depleted CNS retained some macrophage-like properties with respect to cell soma size, process length and arborisation, but were altered in their functionality in a similar fashion as endogenous microglia, when in close proximity to Aβ plaques. This indicates an immediate alteration of myeloid cell function upon entering the Aβ-rich environment of AD-like mice, irrespective of the origin of these cells. To substantiate this apparent functional alteration in myeloid cells in the presence of Aβ plaques, we thought to analyse the response of endogenous microglia vs. PDMCS to a focal laser-induced tissue lesion, which is known to elicit a robust response by endogenous microglia^35^.

### Myeloid cells in the Aβ-rich environment irrespective of their origin show an attenuated response to focal injury

**Figure 4:**
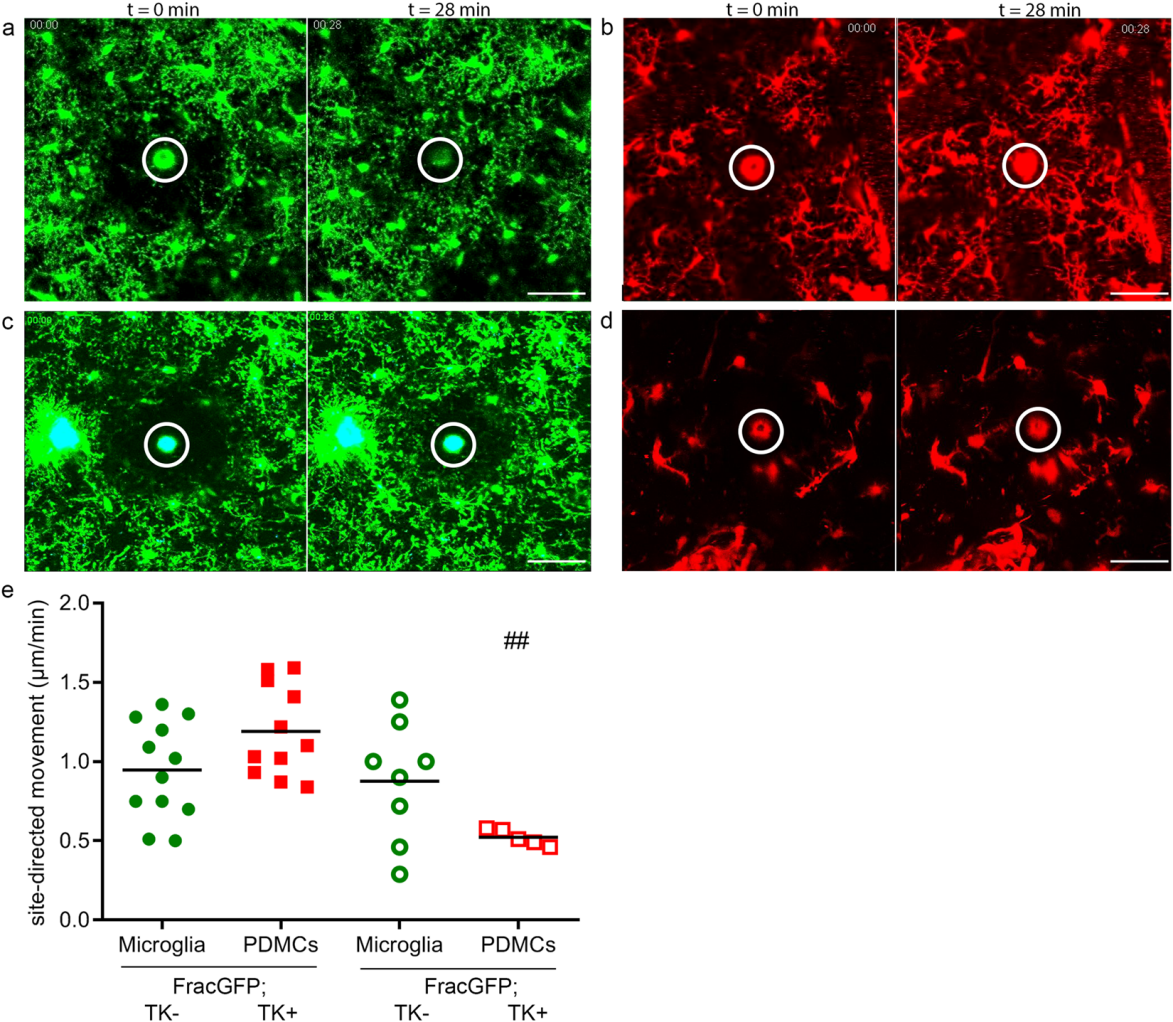
Myeloid cells in the Aβ-rich environment irrespective of their origin show an attenuated response to focal injury. (**a**–**d**) Laser lesions were performed max. 12 days after surgery. Images from first and final time point (t) after lesion infliction illustrating the movement of the myeloid cells and their processes towards the lesion); scale bar = 100 µm. (**a**) Frac-GFP;TK− mice (See also Supplementary Movie 3) (**b**) Frac-GFP;TK+ mice (See also Supplementary Movie 4) (**c**) Frac-GFP;APP+;TK− mice (See also Supplementary Movie 5) (**d**) Frac-GFP;APP+;TK+ mice (See also Supplementary Movie 6). (**e**) Quantification of site-directed process motility. Frac-GFP;TK−: 0.95 ± 0.3 μm/min; Frac-GFP;TK+ mice: 1.19 ± 0.3 μm/min; Frac-GFP;APP+;TK− 0.88 ± 0.4 μm/min; Frac-GFP;APP+;TK+ mice: 0.52 ± 0.1 μm/min. One dot represents one laser lesion, n = 5 animals per genotype. Comparison between groups by 1-way ANOVA with Tukey’s post-hoc test; ^##^p < 0.01. ^#^ denotes significant differences between cell types in WT vs AD environment.

To induce a focal injury, a laser lesion was performed as previously described^6,9,35^. The site-directed motility of the processes of the respective myeloid cells in response to the injury was assessed using ImageJ. In the non-AD setting endogenous microglia readily responded to the local injury and sealed up the defect quickly (Fig. 4a,e, Supplementary Movie 3). The PDMCs display a trend to a slightly faster response, correlating with their faster process extension speed when compared to endogenous microglia (Fig. 4b,e, Supplementary movie 4). In the AD-like setting, however, we observed an attenuated response of endogenous microglia to the injury (Fig. 4c,e, Supplementary Movie 5), in line with our previous findings in a similar setting^9^. PDMCs reacted similar as the endogenous microglia (Fig. 4d,e, Supplementary movie 6), indicating an alteration in the functional state of the PDMCs, despite the fact that the newly recruited cells were only exposed to the Aβ-rich environment for a relatively short period of time.

## Discussion

Microglia cells have been recognized as an important component of the innate immune response to AD neuropathological hallmarks^36^, but there is still a big debate as to whether they exacerbate disease progression by gaining toxic functions or if they rather lose protective functions during the progression of AD. We have previously shown that Aβ deposition in mouse models mimicking certain aspects of AD leads to an impairment of microglia^9^ accelerating a decline in microglia functions observed during aging^8^. The previously described impairment of microglia phagocytic functions in the presence of Aβ deposits^9^ can serve as an explanation for the findings that depletion of microglia did not affect the Aβ burden in two different AD-like mouse models with ablated microglia^28,37^. Despite the fact that microglia are seemingly not capable of degrading Aβ plaques, recent studies have demonstrated that microglia are important for modulating the toxicity of deposited Abeta^21,38,39^, highlighting a protective function of microglia in AD. The phenotype of microglia during the progression of AD-like pathology in mice has been studied extensively resulting in the demonstration that AD-associated microglia express more macrophage-like surface molecules and lose typical markers of endogenous microglia^10,40,41^. This has led to speculations that PDMCs significantly contribute to the innate immune response in AD^42^, but more sophisticated experiments show that there is only minimal if any contribution of infiltrating PDMCs^20–22^ during the natural course of disease. Our data in the present study, monitoring endogenous microglia in a mouse model of AD-like pathology utilizing long-term 2-photon microscopy^29^ are in line with the latter notions; we are able to unequivocally demonstrate that endogenous microglia in AD-like mice adopt macrophage-like properties with reduced soma size, reduced process branchpoints as well as a reduced sum of process length when in close proximity to Aβ deposits. Furthermore, in line with our previous findings, we show that this phenotypic shift is associated with impairment of microglia functions including their natural capacity to respond to local injury^9^. To overcome this disease-associated loss of microglia integrity, the supplementation or exchange of the chronically stimulated and functionally impaired microglia pool with PDMCs has been proposed as a therapeutic strategy for a variety of CNS conditions^43^ including AD^19,23^. To study this in a controlled experimental setting, we and others have recently developed a model that allows for an inducible depletion of >90% of endogenous microglia and repopulation of the microglia-depleted CNS with PDMCs^25,26,32^. Surprisingly, this replacement of functionally impaired microglia with PDMCs in AD-like mice did not alter the Aβ plaque burden after short term (ref. ^25^ and present study) and long term observation^26^. Following up on this finding, we monitored the process of microglia depletion and CNS repopulation by PDMCs by 2-photon microscopy in the present study. Analysing morphologic and functional parameters of the newly recruited PDMCs, the newly recruited cells appear to have a faster response to local injury than endogenous microglia in the non-AD environment. A similar difference in response to local injury has been shown before^44^. In the Aβ-rich environment, however, the PDMCs show an even stronger functional impairment in response to local injury than endogenous microglia. This functional impairment was apparent, although the newly recruited cells were residing in the Aβ-rich CNS environment for a relatively short period of time, arguing for an immediate effect of the diseased environment on myeloid cell functions rather than an effect of chronic, long-term exposure. Although the exchange of endogenous microglia with PDMCs per se did not affect the Aβ plaque burden^25,26^, we had previously demonstrated that the newly recruited cells appear to be more responsive to stimulatory triggers promoting a mild increase in Aβ clearing capacities^25^. While our present data demonstrate functional impairment of PDMCs in a comparable fashion as endogenous microglia upon entering the Aβ-rich CNS environment, we also show that these cells retain some macrophage-like properties, serving as a possible explanation for the at least mildly enhanced responsiveness to stimulation we had observed in a previous study^25^. These results are in line with a recent study, where a single-cell sequencing approach showed that PDMCs maintain a unique transcriptional and functional identity after engrafting into the brain^44^.

While the depletion of the entire microglia pool and repopulation by PDMCs is a good model to study myeloid cell dynamics in controlled experimental settings, it is most likely not a feasible therapeutic approach for patient populations. Nevertheless, recent research efforts into the recruitment of peripheral macrophages to the CNS have led to promising approaches to achieve significant engraftment of peripheral myeloid cells in the brain without the need for opening the blood brain barrier or depletion of endogenous microglia^45^. These efforts and the obvious importance of the innate immune response in AD progression^46^ have pushed myeloid cell-based approaches into the spotlight of viable therapeutic options. Our finding in the present study, namely that newly recruited PDMCs undergo similar functional changes as endogenous microglia upon entering the Aβ-rich CNS environment, highlights the importance of a better understanding of the interaction between myeloid cells and Aβ deposits, as well as the mechanisms of myeloid cell activation in the Aβ-rich CNS environment in order to make use of the potential of myeloid cell-based therapies for AD.

## Materials and Methods

### Mice

The following transgenic mouse lines were used in the study: APPPS1 mice^34^, kindly provided by Matthias Jucker (Tübingen), CX3CR1-GFP mice^47^, kindly provided by Helmut Kettenmann (Berlin), and CD11b-HSVTK^+/−^ mice^24^. CX3CR1-GFP^+/−^ males were crossed to CD11b-HSVTK^+/−^ females to generate CX3CR1-GFP^+/−^;CD11b-HSVTK^+/−^ (Frac-GFP;TK+) and (Frac-GFP;TK−) mice. Female APPPS1^+/−^;CD11b-HSVTK^+/−^ mice were crossed to CX3CR1-GFP^+/−^ males to generate CX3CR1-GFP^+/−^;APPPS1^+/−^;CD11b-HSVTK^+/−^ (Frac-GFP;APP+;TK+) and (Frac-GFP;APP+;TK−) mice. Experimental mice and respective control mice were littermates and of mixed gender. Animals were group-housed in standard cages under pathogen-free conditions on a 12 hour light/dark cycle with food and water ad libitum. All animal experiments were performed in accordance to the national animal protection guidelines approved by the regional offices for health and social services in Berlin (LaGeSo Berlin)^−^.

#### Bone marrow chimerism

Bone marrow chimeric mice were generated by exposing 4 months old mice to 10 Gray whole-body irradiation (see also Fig. 1a), followed by intravenous injection of with 10^7^ bone marrow cells isolated from ROSA26-tandem red fluorescent protein (tdRFP) reporter mice^27^, kindly provided by Jana Glumm (Berlin). Subsequently, mice were treated with antibiotics (0.01% Enrofloxacin, Baytril) for four weeks.

#### Microglia depletion and PDMC repopulation

Four weeks after bone marrow transplantation a mini-osmotic pump (model 2001 cat.no. 0000292, Alzet) containing 2.5 mg Cymeven/mL in artificial cerebrospinal fluid (aCSF) was implanted on the right hemisphere to achieve microglia depletion as described before^28^. During the same surgery, a cranial window was placed on the left hemisphere, as described before^29,30^, surrounded by a custom-made titanium ring for 2-photon imaging. After 10 days, the mini-osmotic pump (model 2001) was replaced by a long term mini-osmotic pump (model 2004 cat.no. 0000298, Alzet) containing 2.5 mg Cymevene/ml in aCSF, without disturbing the cannula. After four weeks the 2004 model pump was removed.

#### 2-photon imaging

2-photon imaging was performed using an adapted TrimScope II microscope (LaVision Biotec, Bielefeld, Germany) previously described^48^. Briefly, to excite eGFP in CX3CR1 GFP mice as well as injected Methoxy X04 we used a Ti:Sa laser (Ultra II, Coherent, Dieburg, Germany) at 850 nm. tdRFP in cells of ROSA26-tandem red fluorescent protein reporter mice was excited by an optical parametric oscillator at 1110 nm. Both laser beams were focused by an IR-coated 20x dipping objective lens (NA = 0.95, WD = 2 mm, Olympus, Hamburg, Germany) for intravital imaging. The emitted light was analysed using dichroic mirrors and interference filters and detected using photomultiplier tubes (H7422-40; Hamamatsu, Japan). We used the following detection channels: eGFP - 525/50 nm, Methoxy X04 - 460/60 nm and tdRFP - 593/40 nm. At least one day prior to each imaging session, APPPS1^+/−^ animals were injected intraperitoneally with 10 mg/kg Methoxy X04 to visualize Aβ plaques^49^. For 2-photon imaging, a custom-build head fixation apparatus that allows precise relocation of imaging positions over weeks was installed on the motorized xy stage of the 2-photon microscope. The microscope coordinates were set to origin (x = 0, y = 0) at a defined fluorescently marked label on a localization ring. Mice were fixed into the head fixation apparatus with a custom-build titanium ring. Using the automated XY locator function of ImSpector Pro (LaVision Biotec), the same position was used every imaging session with minimal manual fine tuning using either blood vessels or the amyloid beta plaque landscape as reference. During each imaging session three fields of view (300 × 300 μm²) per mouse were imaged at 400 Hz with constant laser power of ≈10 mW and a resolution of 512 × 512 pixels. Under these conditions no effects of phototoxicity are to be expected. A z-stack of approximately 70 μm thickness was recorded, starting at a cortex depth of minimal 40 μm, in 2 μm step size. All fields of view were imaged every minute for a minimum of 30 minutes.

#### Analysis

All recorded imaging videos were registered using the “Correct 3D drift” Plugin of ImageJ to correct for translational drifts^50^. Further analysis was performed in Imaris 7.0; Bitplane, Zurich, Switzerland.

For soma size and sphericity, the surfaces of cells were detected with the surface reconstruction algorithm of Imaris. All cell somas of one field of view were detected automatically in a batch, subsequently each movie was manually filtered for high quality detected somas. For soma size and soma sphericity, only the mean values for all somas in one field of view from the first minute of each imaging time point were used.

For the analysis of cell proximity to plaques, cells were detected using the spot recognition algorithm of Imaris at the first minute of each imaging time point. Spots were manually checked to exclude double labelling of one cell and to ensure detection of every soma present in the imaging volume. Plaques were detected at the same time point using the surface reconstruction algorithm. The xyz coordinates of spots and plaques were exported in VRML (Virtual Reality Modeling Language) format (http://gun.teipir.gr/VRML-amgem/spec/index.html) and the Euclidian distances between cells and plaques were measured for every detected cell with a custom-written algorithm using Python scripting in Blender 2.7 (https://www.blender.org). Cell to plaque distance <10 µm was considered in close proximity to plaques.

The cell’s filament tree was reconstructed using the filament detection algorithm of Imaris for each cell individually. Only cells not extending past the imaged volume were used for analysis and each cell was double-checked manually after automated tracking.

To quantify cytoplasmic process extension and retraction, we used maximum intensity projections from the first recorded time point and five minutes later. For each imaging time point three fields of view with high quality resolution and signal were chosen. Five extensions and retractions of two to five cells were randomly quantified with ImageJ as described before^6^.

#### Laser lesion

For laser induced lesions, we applied a laser power of ≈200 mW for three seconds in a point scan mode, as described before^6,35^. Imaging was performed right after lesion induction as described above. Quantification of laser lesion-induced microglial reaction was performed in ImageJ using maximum intensity projections for every minute of the imaged movie. The distance of microglial cytoplasmic processes to the autofluorescent lesion was measured every second minute.

#### Immunohistochemistry

After the last imaging session, the animals were sacrificed using CO2, and perfused with PBS. The brain was harvested and fixed in 4% paraformaldehyde. Free floating sections of 40um were cut with a cryostat, and stained with anti-Iba1 (1:500, Wako, 019-19741), anti-GFP (1:500, Abcam, AB290), anti-RFP (1:200, Rockland, 600-406-379) to visualize myeloid cells, with anti-CD68 (1:100, AbD Serotec, MCA1957) for activation, or for aβ with 4G8 (1:500, Covance, Sig39220-500). Stereology was performed using a Stereo Investigator system (MicroBrightField) and DV-47d camera (MicroBrightField) mounted on a BX53 microscope (Olympus). Stereological quantification of Iba1+, GFP+ and RFP+ cells were performed using the Optical Fractionator method (MicroBrightField). Quantification of 4G8 + plaques was performed using the Area Fraction fractionator method (MicroBrightField). Percentage of activated myeloid cells around plaques was analysed with ImageJ from maximum intensity images compiled from a stack, taken with a Leica TCS SP5 confocal laser scanning microscope controlled by LAS AF scan software (Leica Microsystem, Wetzlar, Germany).

#### Statistics

All values are mentioned as mean ± standard deviation (SD). T-tests were used to compare two groups. Comparisons between more than two groups were done either by a one-way or by a two-way analysis of variance (ANOVA) with Tukey’s or Sidak’s multiple comparisons post-test. P-values of less than 0.05 were considered to be significant. Statistical tests are mentioned in the figure legends where of relevance.

## Supplementary information

Supplementary Information.

Supplementary Movie 1

Supplementary Movie 2 

Supplementary Movie 3

Supplementary Movie 4

Supplementary Movie 5

Supplementary Movie 6

## Data Availability

All data generated or analyzed during this study are included in this published article (and its Supplementary Information files).
