# Supplementary material for: The Amyloid-beta rich CNS environment alters myeloid cell functionality independent of their origin
Source: Sci Rep. 2020 Apr 28;10:7152. doi: 10.1038/s41598-020-63989-3 (PMC7189379; doi:10.1038/s41598-020-63989-3)
Supplement: Supplementary file 1 — Supplementary Information. [file 41598_2020_63989_MOESM1_ESM.pdf]

Supplemental Data

The Amyloid-beta rich CNS environment alters myeloid cell functionality independent of their origin

Authors: Natalia Drost[1,§], Judith Houtman[1,2§], Zoltán Cseresnyés[3,4], Raluca Niesner[3,5], Jan-Leo Rinnenthal[1,6], Kelly R Miller[1,7], Stefan Prokop[1,8,9,10,&], Frank L Heppner[1,,11,12,13,&,*]

Affiliations:

[1] Department of Neuropathology, Charité – Universitätsmedizin Berlin, corporate member of Freie Universität Berlin, Humboldt-Universität zu Berlin, and Berlin Institute of Health, 10117 Berlin, Germany

[2] Present address: German Center for Neurodegenerative Diseases (DZNE) Dresden, 01307 Dresden, Germany

[3] Deutsches Rheuma-Forschungszentrum Berlin, a Leibniz Institute, Charitéplatz 1, 10117 Berlin, Germany

[4] Present address: Applied Systems Biology, Leibniz Institute for Natural Product Research and Infection Biology – Hans Knöll Institute, Jena, Germany

[5] Veterinary Medicine, Freie Universität, Berlin, Oertzenweg 19b, 14163 Berlin, Germany

[6] Present address: Department of Pathology, Sana Klinikum Offenbach, 63069 Offenbach, Germany

[7] Present address: Nanostring Technologies, Seattle, WA, USA.

[8]Present address: Department of Pathology, University of Florida, Gainesville, FL, United States

[9] Present address: Center for Translational Research in Neurodegenerative Disease, University of Florida, Gainesville, FL, United States

[10] Present address: Fixel Institute for Neurological Diseases, University of Florida, Gainesville, FL, United States

[11] Cluster of Excellence, NeuroCure, Charitéplatz 1, 10117 Berlin, Germany

[12] Berlin Institute of Health (BIH), 10117 Berlin, Germany

[13] German Center for Neurodegenerative Diseases (DZNE) Berlin, 10117 Berlin, Germany

[§] authors contributed equally

[&] shared senior authorship

[*] Corresponding author, frank.heppner@charite.de

Figure S1

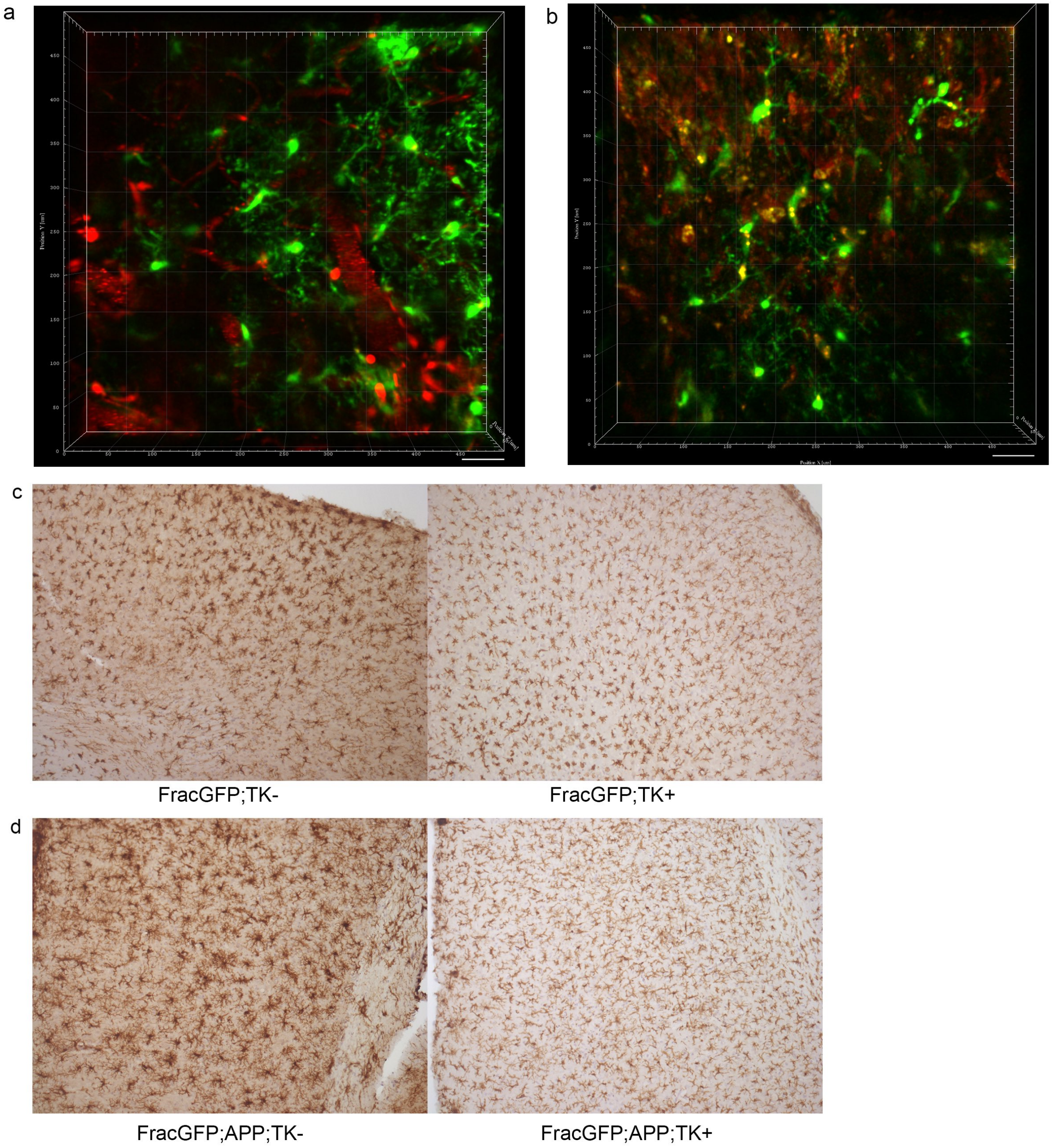

Figure S1. Microglia morphology and distribution. (a, b) 3D images created with Imaris representing microglia (a) and PDMC (b) morphology at day 6 of imaging. (c, d) Iba1+ cell distribution and morphology throughout the cortex in the different animal models used.

Figure S2

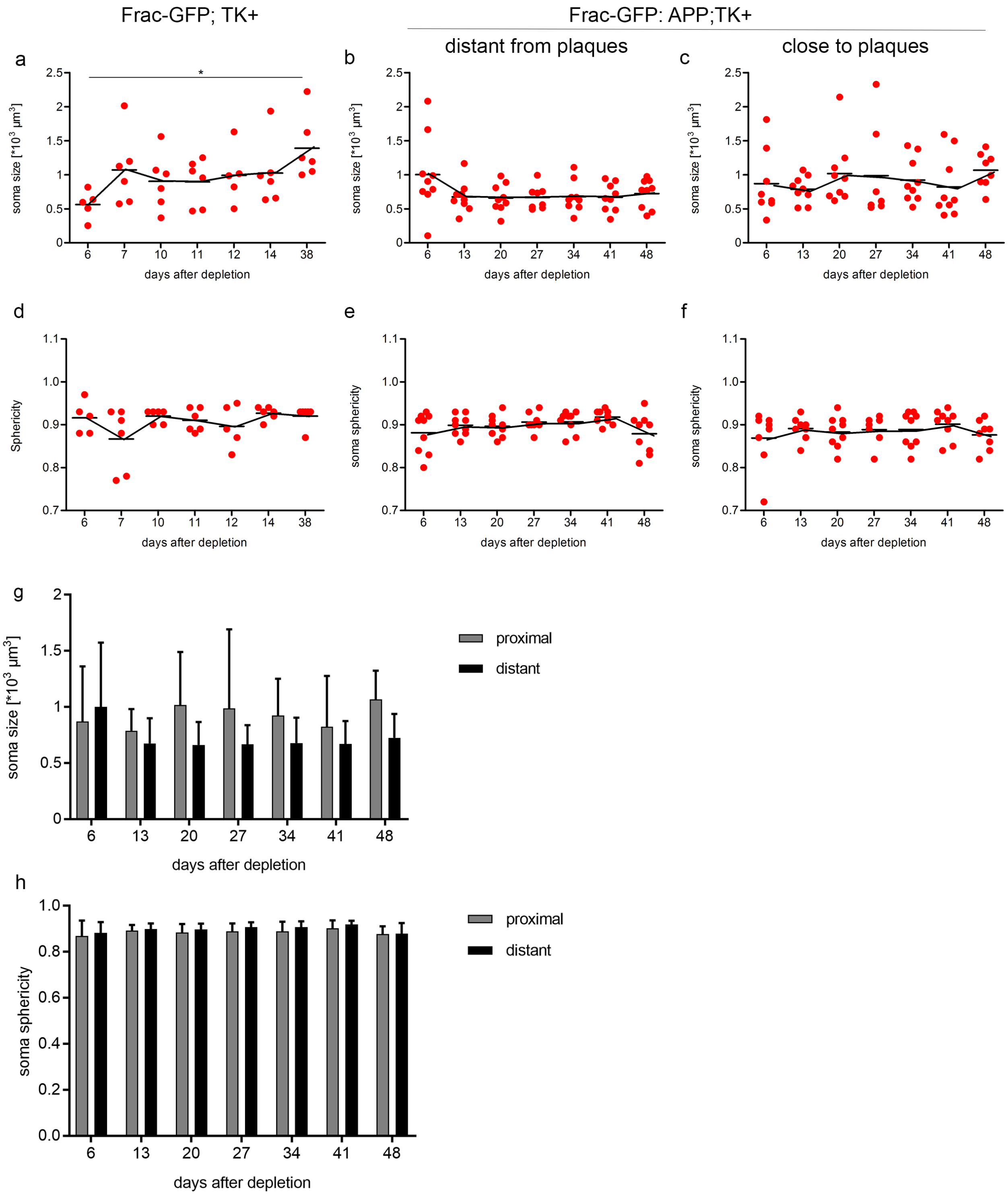

Fig. S2 Soma morphology assessment over time. Two morphological parameters assessed for the PDMCs of each of the indicated genotypes for each time point after surgery: (a-c) soma size, (d-f) soma sphericity. Statistics: 1-way ANOVA, with Tukey's post-hoc test. * $p<0.05$. (g, h) comparison of proximal and distant PDMCs in APP animals over time. Statistics: 2-way ANOVA.

Figure S3

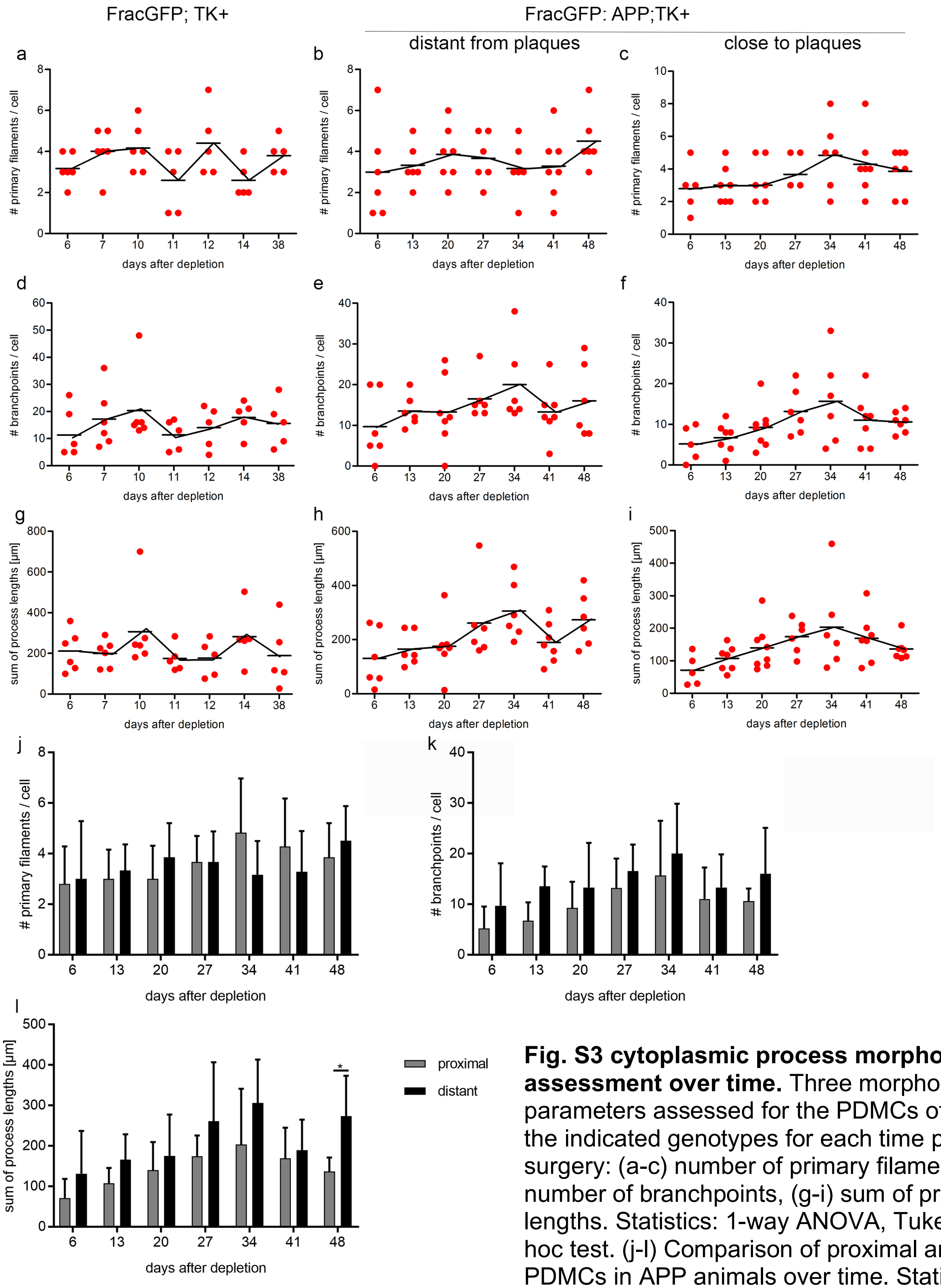

Fig. S3 cytoplasmic process morphology assessment over time. Three morphological parameters assessed for the PDMCs of each of the indicated genotypes for each time point after surgery: (a-c) number of primary filaments, (d-f) number of branchpoints, (g-i) sum of protrusion lengths. Statistics: 1-way ANOVA, Tukey's post-hoc test. (j-l) Comparison of proximal and distant PDMCs in APP animals over time. Statistics: 2-way ANOVA. * $p < 0.05$.

Figure S4

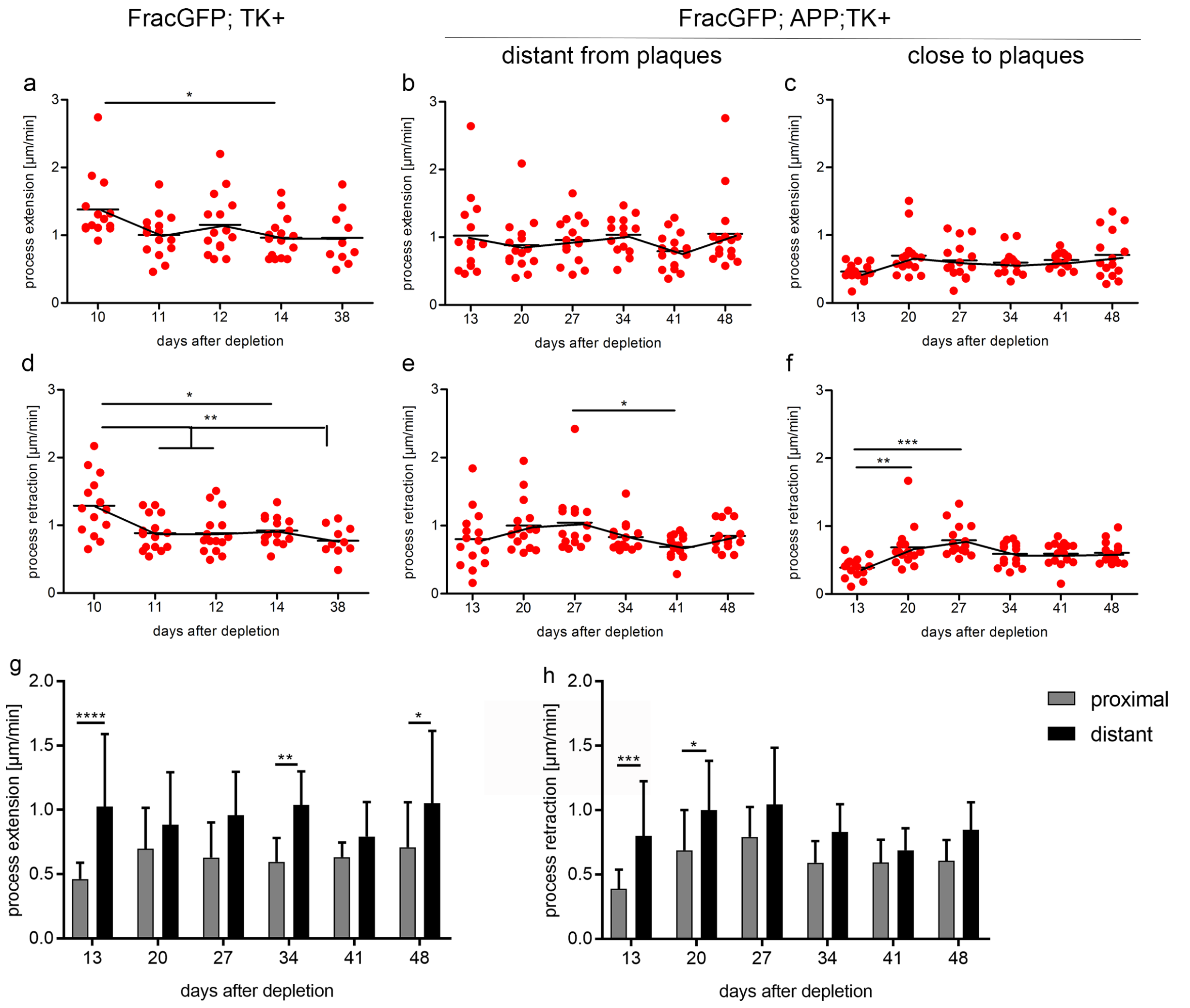

Fig. S4 cytoplasmic process activity assessment over time. Both process extension (a-c) as well as process retraction (d-f) were assessed for the PDMCs of each of the indicated genotypes for each time point after surgery. Statistics: 1-way ANOVA, Tukey's post-hoc test. * $p<0.05$, ** $p<0.01$, *** $p<0.001$. (g,h) Comparison of proximal and distant PDMCs in APP animals over time. Statistics: 2-way ANOVA. * $p < 0.05$, *** $p<0.001$, **** $p<0.0001$.

Supplementary Movie 1. Day 6 after surgery.

Animation of the 3D image of a field of view 6 days after surgery, displaying homeostatic GFP+ microglia.

Supplementary Movie 2. Day 24 after surgery.

Animation of the 3D image of a field of view 24 days after surgery, displaying homeostatic GFP+ microglia.

Supplementary Movie 3. Microglia response to laser lesion in FracGFP;TK- animals

Time lapse of 28 minutes of the microglia response to a laser lesion in FracGFP;TK- animals.

Supplementary Movie 4. PDMC response to laser lesion in FracGFP;TK+ animals

Time lapse of 28 minutes of the PDMC response to a laser lesion in FracGFP;TK+ animals.

Supplementary Movie 5. Microglia response to laser lesion in FracGFP;APP+;TK- animals

Time lapse of 28 minutes of the microglia response to a laser lesion in FracGFP;APP;TK- animals.

Supplementary Movie 6. PDMC response to laser lesion in FracGFP;APP+;TK+ animals

Time lapse of 28 minutes of the PDMC response to a laser lesion in FracGFP;APP;TK+ animals.
